# AMPK activation inhibits the functions of myeloid-derived suppressor cells (MDSC): impact on cancer and aging

**DOI:** 10.1007/s00109-019-01795-9

**Published:** 2019-05-25

**Authors:** Antero Salminen, Anu Kauppinen, Kai Kaarniranta

**Affiliations:** 1 Department of Neurology, Institute of Clinical Medicine, University of Eastern Finland, P.O. Box 1627, FI-70211 Kuopio, Finland; 2 School of Pharmacy, Faculty of Health Sciences, University of Eastern Finland, P.O. Box 1627, FI-70211 Kuopio, Finland; 3 Department of Ophthalmology, Institute of Clinical Medicine, University of Eastern Finland, P.O. Box 1627, FI-70211 Kuopio, Finland; 4 Department of Ophthalmology, Kuopio University Hospital, P.O. Box 100, FI-70029 Kuopio, Finland

**Keywords:** Aging, Immunosuppression, Immunosenescence, Immunotherapy, Longevity, Trained immunity

## Abstract

AMP-activated protein kinase (AMPK) has a crucial role not only in the regulation of tissue energy metabolism but it can also control immune responses through its cooperation with immune signaling pathways, thus affecting immunometabolism and the functions of immune cells. It is known that AMPK signaling inhibits the activity of the NF-κB system and thus suppresses pro-inflammatory responses. Interestingly, AMPK activation can inhibit several major immune signaling pathways, e.g., the JAK-STAT, NF-κB, C/EBPβ, CHOP, and HIF-1α pathways, which induce the expansion and activation of myeloid-derived suppressor cells (MDSC). MDSCs induce an immunosuppressive microenvironment in tumors and thus allow the escape of tumor cells from immune surveillance. Chronic inflammation has a key role in the expansion and activation of MDSCs in both tumors and inflammatory disorders. The numbers of MDSCs also significantly increase during the aging process concurrently with the immunosenescence associated with chronic low-grade inflammation. Increased fatty acid oxidation and lactate produced by aerobic glycolysis are important immunometabolic enhancers of MDSC functions. However, it seems that AMPK signaling regulates the functions of MDSCs in a context-dependent manner. Currently, the activators of AMPK signaling are promising drug candidates for cancer therapy and possibly for the extension of healthspan and lifespan. We will describe in detail the AMPK-mediated regulation of the signaling pathways controlling the expansion and activation of immunosuppressive MDSCs. We will propose that the beneficial effects mediated by AMPK activation, e.g., in cancers and the aging process, could be induced by the inhibition of MDSC functions.

## Introduction

AMP-activated protein kinase (AMPK) acts at the nexus of the regulation of energy metabolism and immune responses, with involvements in the activities of both innate and adaptive immunity [1, 2]. AMPK activation regulates cellular immunity in cooperation with immune signaling pathways and controlling energy metabolism which consequently affects the activation of immune cells. Since AMPK signaling maintains cellular homeostasis, it is not surprising that its function is disturbed in many chronic diseases [3, 4]. However, it seems that the regulation of AMPK signaling is a complex and context-dependent process which can generate even opposite effects in chronic diseases, e.g., in tumorigenesis [5]. It is known that AMPK activation can suppress inflammatory responses by inhibiting the functions of two major immune signaling pathways, i.e., nuclear factor-κB (NF-κB) and signal transducer and activator of transcription (STAT) pathways [6, 7]. Interestingly, the same signaling pathways control the expansion and activation of myeloid-derived suppressor cells (MDSC) which induce an immunosuppressive microenvironment around tumors and thus allow tumor cells to escape from immune surveillance [8, 9]. The numbers of MDSCs also increase with aging concurrently with the immunosenescence associated with a low-grade inflammation [10, 11]. There is convincing evidence that AMPK activation can inhibit tumor growth and even extend the healthspan and lifespan (see below). We will examine in detail the repressive effects of AMPK signaling on the expansion of MDSCs and their activation and functions in inflamed tissues, such as tumors and aging tissues.

### AMPK controls energy metabolism and immune responses

AMPK signaling not only has a crucial role in energy metabolism but it also undertakes a close crosstalk with other signaling pathways in order to maintain tissue homeostasis in the face of diverse stresses [12, 13]. AMPK activation stimulates energy production through glucose and fatty acid oxidation, whereas it inhibits anabolic processes, e.g., glycogen synthesis, gluconeogenesis, as well as fatty acid and cholesterol syntheses. Nonetheless, there exist distinct tissue-specific differences in the functions of AMPK. The major activators of AMPK are (i) liver kinase B1 (LKB1), activated by AMP and (ii) Ca^2+^/calmodulin-dependent protein kinase β (CaMKKβ), stimulated by increased Ca^2+^ concentration. There are a number of downstream targets for AMPK signaling, typically the enzymes controlling energy metabolism, cell growth, and autophagy. AMPK activation also regulates the functions of many transcription factors, in either a direct or indirect manner. In addition to energy metabolism, AMPK signaling controls the proteostasis of cells, i.e., it inhibits mammalian target of rapamycin (mTOR) thus reducing protein synthesis but in contrast, it activates autophagic degradation via the stimulation of ULK1 [14]. Accordingly, AMPK signaling has a crucial role in tumor growth [5, 15] and the aging process [16, 17].

There is convincing evidence that AMPK activation prevents inflammatory responses through the inhibition of pro-inflammatory signaling pathways [1, 6]. For instance, the activation of AMPK inhibits the function of the NF-κB system as well as JAK-STAT signaling, both of which are major pathways of inflammatory and immune responses [6, 7]. In macrophages, the activation of AMPK promotes the polarization of M1 pro-inflammatory macrophages towards the M2 anti-inflammatory phenotype [18]. Moreover, AMPK activation regulates the signaling of the anti-inflammatory cytokine, IL-10, in macrophages, e.g., enhancing the IL-10-induced suppression of LPS-stimulated cytokine production [19]. Ishii et al. [20] demonstrated that the activation of AMPK suppressed the proliferation of macrophages induced by oxidized low-density lipoprotein (Ox-LDL). They also reported that AMPK activation inhibited the Ox-LDL-induced expression of granulocyte-macrophage colony-stimulating factor (GM-CSF). This is an important observation since GM-CSF is a potent inducer of MDSC generation in the bone marrow [9, 21]. Several studies have also demonstrated that the activation of AMPK has an important role in the differentiation and functions of T lymphocytes by regulating their energy metabolism [2, 22]. These observations clearly indicate that AMPK signaling controls the balance between energy metabolism and immune responses.

### MDSCs are inducers of immunosuppression

By definition, MDSCs are immature myeloid cells which display immune suppressive properties against adaptive and innate immunity [8, 9]. MDSCs originate from hematopoietic stem cells through the lineage of common myeloid progenitors in the bone marrow. Several pathological conditions in the body, e.g., tumorigenesis and inflammatory disorders, are known to trigger emergency myelopoiesis which stimulates the proliferation of immature myeloid cells (IMC), the progenitors of MDSCs, in the bone marrow and consequently IMCs can migrate into extramedullary sites, e.g., spleen and lymph nodes. The emergency signaling originating from tumors and inflamed tissues stimulates the differentiation of IMCs into MDSCs and promotes their expansion. For instance, colony-stimulating factors, e.g., GM-CSF and G-CSF, stimulate emergency myelopoiesis and thus trigger the differentiation and expansion of MDSCs through the regulation of STAT signaling pathways [23, 24]. The chemotaxis of MDSCs into tumors and inflamed tissues is driven by several chemokines, e.g., CC ligand 2 (CCL2), CXC chemokine ligand 2 (CXCL2), and CXCL8/IL-8. In inflamed tissues, the microenvironmental conditions control the proliferation of MDSCs and the induction of their immune suppressive activities [8, 10, 25, 26]. Several cytokines, e.g., IL-1β, IL-6, IL-18, and TNF-α, as well as some alarmins, such as HMGB1 and S100A8 and A9, are potent enhancers of immunosuppressive properties of MDSCs. These inflammatory mediators stimulate the expression immunosuppressive genes through distinct transcription factors, e.g., STATs, NF-κB, and HIF-1α pathways. There are two different phenotypes of MDSCs, i.e., monocytic and granulocytic MDSCs, which not only display differences in their generation but they also possess distinct immunosuppressive phenotypes [25, 27, 28]. However, the functional differences need to be clarified since most studies investigating MDSCs have not differentiated between these two phenotypes.

MDSCs are immunosuppressive cells which suppress mainly the functions of T and B lymphocytes but they can also inhibit the innate immunity responses of myeloid cells [8, 27, 29]. MDSCs possess the potent immune suppressive armament with different inhibitory mechanisms, as described in detail elsewhere [8, 27, 30]. MDSCs secrete several anti-inflammatory cytokines, such as TGF-β and IL-10; not only do these factors inhibit immune responses but they are also able to enhance the functions of other immunosuppressive cells, e.g., regulatory T cells (Tregs) and B cells (Bregs) [31, 32]. For instance, the presence of TGF-β and IL-10 inhibits the proliferation and functions of T and B cells [31, 33]. Moreover, these cytokines can induce the alternative polarization of proinflammatory M1 macrophages towards the anti-inflammatory M2 phenotype [31]. MDSCs also secrete reactive oxygen species (ROS) and thus suppress the functions of immune cells [30, 34]. The activation of MDSCs induces the expression of NADPH oxidase (NOX2) and inducible nitric oxide synthase (iNOS) which generates different ROS compounds and nitric oxide (NO). It is known that the product of H_2_O_2_ and NO, i.e., peroxynitrite (ONOO^−^), is able to nitrate the tyrosine residues of T cell receptor (TCR) after which the receptor no longer recognizes antigen peptides and thus the TCR signaling pathway is inhibited [35]. MDSCs also promote immunosuppression since they induce the expression of amino acid metabolizing enzymes leading to a shortage of critical amino acids in the inflammatory microenvironment [36]. Arginase 1 (ARG1) and indoleamine 2,3-dioxygenase (IDO) catabolize arginine and tryptophan, respectively, which inhibit protein synthesis and subsequently, impair the functions of immune cells. MDSCs can also suppress immune functions in a contact-dependent manner through the so-called checkpoint receptors. The activation of MDSCs stimulates the expression of programmed death-ligand (PD-L1) receptor which binds to the PD-1 receptor, e.g., in T cells and macrophages [37, 38]. Currently, the immunotherapy of PD-L1 blockade is viewed as a promising treatment in many cancers [39].

The activation of emergency myelopoiesis not only generates proinflammatory myeloid cells but it also stimulates the production of immunosuppressive MDSCs [9, 40, 41]. In inflamed tissues, MDSCs have an important role in the resolution of inflammation [41, 42]. In particular, the secretion of IL-10 and TGF-β increases the phagocytic activity of macrophages. Saiwai et al. [42] demonstrated that MDSCs enhanced the resolution process of acute inflammation after spinal cord injury (SCI) in mice. Infiltrated MDSCs accelerated the removal of hematomas and tissue debris as well as stimulating angiogenesis after SCI. They also reported that the transplanted MDSCs promoted the repair process and the recovery of functional properties in mouse SCI. However, in chronic inflammatory disorders, where the perpetrator has not been removed, e.g., in tumors, chronic infections, autoimmune diseases, and many degenerative diseases, MDSCs suppress the functions of immune cells of both adaptive and innate immunity, thus aggravating the pathological condition. The immunosuppressive state has been associated with different kinds of pathology, e.g., tumors, stroke, and sepsis [27, 43, 44]. Immunosenescence, a phenomenon linked to the aging process, causes many similar alterations appearing in an immunosuppressive state in many inflammatory disorders [11, 45].

### Role of MDSCs in tumor growth and aging process

Over 20 years ago, it was discovered that there was a clear association between the presence of MDSCs, earlier called null cells and veto cells, and tumor growth and the escape of tumor cells from immune surveillance [46]. Subsequently, it was observed that inflammation had a crucial role in the expansion and recruitment of MDSCs into tumors and inflamed tissues. Bunt et al. [47] demonstrated that the inoculation of the IL-1β-promoted 4 T1 mammary carcinoma cells into mice increased the infiltration of MDSCs into tumor sites, whereas the accumulation of MDSCs into tumors was clearly delayed in the IL-1 receptor–deficient mice which also displayed reduced tumor growth. Subsequently, the key role of inflammation has been verified in both tumorigenesis and the MDSC-mediated tumor tolerance [27, 48]. As described above, MDSCs possess different immunosuppressive properties which are able to overcome the antitumor immune reactions mediated by T cells and natural killer (NK) cells. In cooperation with Tregs and tumor-associated macrophages (TAM), MDSCs reduce the proliferation of CD4 and CD8 T cells and NK cells, as well as inhibiting the cytotoxicity of CD8 T and NK cells. Multiple strategies have been developed to inhibit the immune suppressive functions of MDSCs in tumors [49, 50]. For instance, there has been a search for potent inhibitors of the major signaling pathways which regulate the immune suppressive properties of MDSCs. Two STAT3 inhibitors, sunitinib and axitinib, were reported to inhibit the recruitment of MDSCs into tumors and to suppress the functions of T cells [51, 52]. Interestingly, it is known that several phytochemicals which are inhibitors of STAT3 and NF-κB signaling also suppress the expansion and activation of MDSCs and thus enhance antitumor immunity, at least in mice [53]. Given that MDSCs are immature cells, there are some observations that all-trans retinoic acid (ATRA) and β-glucan can induce the maturation of MDSCs and thus enhance antitumor activity [54, 55].

The aging process is associated with chronic low-grade inflammation, a condition which has been called inflammaging [56]. Simultaneously, there are anti-inflammatory processes, accordingly termed anti-inflammaging [57]. Intriguingly, the human immune system is suppressed with aging affecting especially T and B lymphocytes, although clear age-related changes also occur in the functions of innate immunity [58]. Currently, the origin of the immunosenescence is not known although many of the changes are similar to those induced by MDSCs [11]. There is convincing evidence that the numbers of MDSCs substantially increase with aging in the bone marrow, blood, spleen, and peripheral lymph nodes. The age-related expansion of MDSCs has been observed in humans [59, 60] and mice [61–63]. An increased level of MDSCs has also been detected in progeroid mice [63]. The inflammaging process also affects the bone marrow which might induce aberrant myelopoiesis [64]. There is a clear increase in myelopoiesis with aging [65] which could be induced by the TGF-β, secreted from MDSCs, since TGF-β signaling enhances the myeloid differentiation of mouse hematopoietic stem cell clones [66]. It seems that the expansion of MDSCs with aging might not only promote immunosenescence but also induce age-related harmful bystander effects in host tissues via the secretion of TGF-β and IL-10 as well as provoking a shortage of distinct amino acids [10].

### AMPK signaling is potential regulator of MDSC functions

**Fig. 1:**
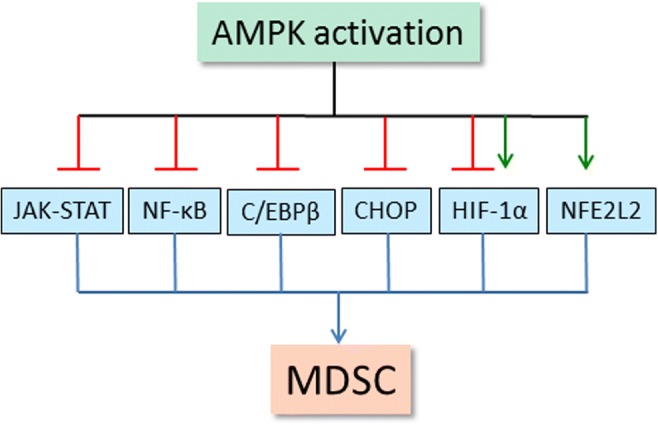
Schematic presentation depicting the signaling connections through which AMPK signaling controls the major activation pathways of MDSCs. A stopper indicates an inhibitory connection and an arrow denotes an activating pathway. Abbreviations: AMPK, AMP-activated protein kinase; C/EBPβ, CCAAT-enhancer-binding protein β; CHOP, C/EBP homologous protein; HIF-1α, hypoxia-inducible factor-1α; JAK, Janus kinase; MDSC, myeloid-derived suppressor cell; NF-κB, nuclear factor-κB; NFE2L2, nuclear factor (erythroid derived 2)-like 2; STAT, signal transducer and activator of transcription

There are several studies indicating that AMPK activators can inhibit the functions of MDSCs and display antitumor activities in many cancers [67–69]. For instance, Trikha et al. [67] demonstrated that OSU-53 treatment, an AMPK activator [70], reduced the numbers of MDSCs in both the spleen and tumors of carcinoma-bearing mice. Simultaneously, administration of OSU-53 increased the numbers of T cells and NK cells in the spleen and tumors. Moreover, treatment with metformin, another AMPK activator, reduced the accumulation of MDSCs into esophageal tumors by inhibiting NF-κB signaling in an AMPK-dependent manner [68]. AMPK signaling can also control energy metabolism and immune processes in cooperation with several other signaling pathways. We will elucidate the association of AMPK activation with the signaling mechanisms which control the expansion and activation of MDSCs. It seems that AMPK signaling is able to inhibit the major immune signaling pathways, such as the JAK-STAT, NF-κB, C/EBPβ, CHOP, and HIF-1α pathways, all of which stimulate the functions of immunosuppressive MDSCs (Fig. 1).

### JAK-STAT3 pathway

**Fig. 2:**
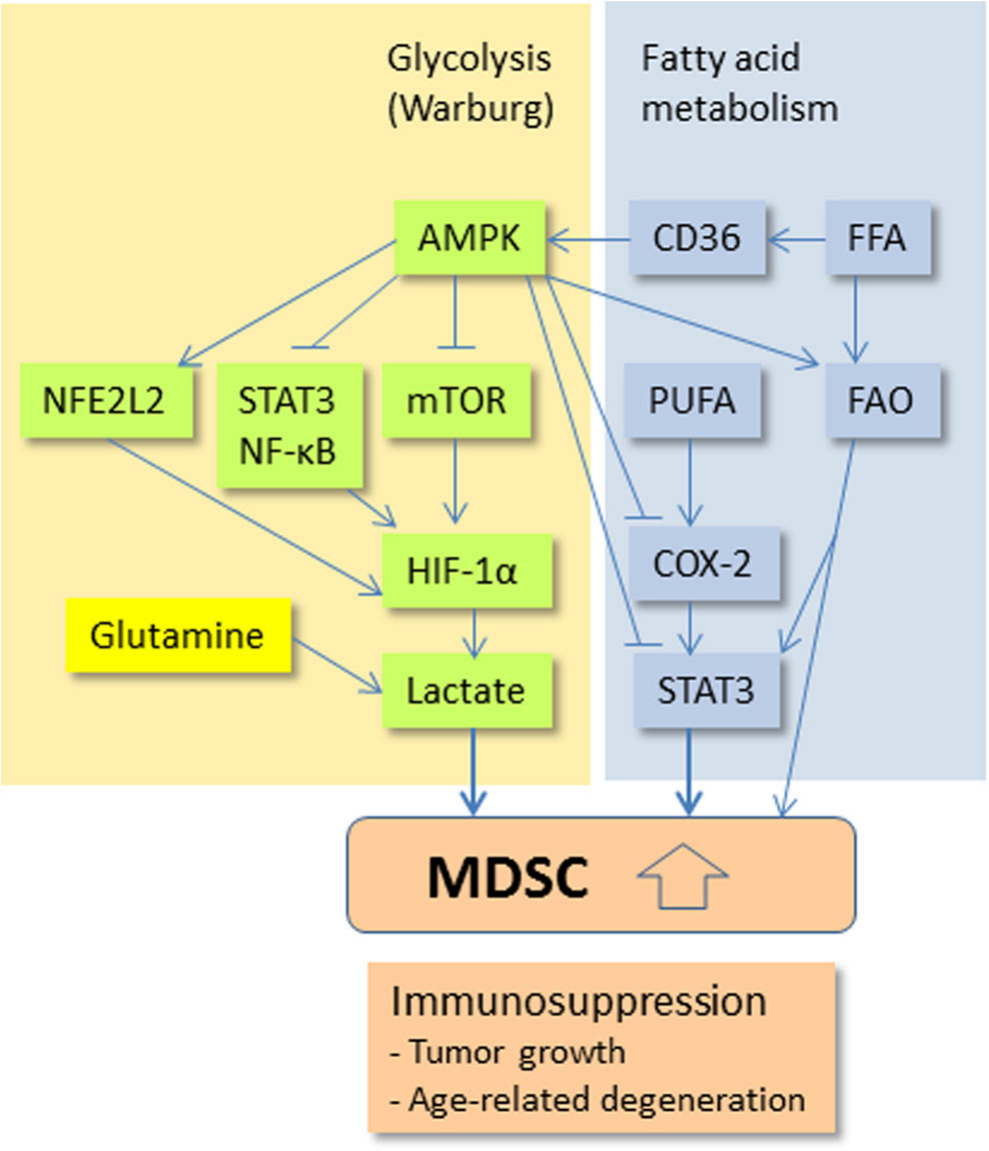
Schematic presentation depicting the energy metabolic pathways through which AMPK controls the activation of MDSCs. The metabolic pathways linked to the aerobic glycolysis and fatty acid metabolism have been described. A stopper indicates an inhibitory connection and an arrow denotes an activating pathway. Increased MDSC activation generates an immunosuppressive state which enhances tumor growth and age-related degeneration in tissues. *Abbreviations:* CD36, cluster of differentiation 36; COX-2, cyclooxygenase-2; FAO, fatty acid oxidation; FFA, free fatty acid; PUFA, polyunsaturated fatty acid. Other abbreviations are as marked in Fig. 1

The JAK-STAT3 signaling pathway has a crucial role in the regulation of immune responses in both the innate and adaptive immune systems [71]. It is known that the STAT3 transcription factor regulates the expression of tens of cytokines and growth factors; thus they can control immune responses and induce the immune escape of tumors. Ko and Kim [24] have reviewed several specific STAT-mediated signaling pathways which induce the generation and activation of MDSCs in mouse and human cancers. In addition to STAT3, other STATs, i.e., STAT1, STAT5, and STAT6, can also enhance the functions of MDSCs. The most important MDSC-related inducers of STAT signaling are GM-CSF, G-CSF, IL-4, IL-6, IL-10, and VEGF. Interestingly, polyunsaturated fatty acids (PUFA) also promoted the expansion of MDSCs via JAK-STAT3 signaling in mice [72] (Fig. 2). Consequently, the JAK-STAT3 pathway stimulated the immune suppressive activities of MDSCs by inducing the expression of ARG1 [73], IDO [74], iNOS [75], and PD-L1 [76]. Waight et al. [23] demonstrated that G-CSF and GM-CSF increased the generation of MDSCs by downregulating the expression of IRF-8 in myeloid progenitors via signaling through STAT3 and STAT5 pathways. Correspondingly, a deficiency of IRF-8 provoked the expansion of the MDSC population in mice. On the other hand, Kumar et al. [77] revealed that the inhibition of STAT3 signaling in mouse MDSCs through the activation of CD45 phosphatase induced the differentiation of MDSCs into TAMs. The inhibition of STAT3 signaling by sunitinib arrested the proliferation and reduced the viability of splenic MDSCs in tumor-bearing mice [78]. There are also several phytochemicals, e.g., curcumin, which have been reported to suppress the functions of MDSCs by inhibiting STAT3 signaling [53]. These observations provide compelling evidence that the JAK-STAT3 pathway has a crucial role in both the differentiation and immunosuppressive properties of MDSCs.

There is substantial evidence that the activation of AMPK signaling inhibits the JAK-STAT pathway and affects many immune responses. For instance, Rutherford et al. [7] revealed that AMPK activation inhibited the activity of Janus kinase 1 (JAK1) by directly phosphorylating the protein on Ser^515^ and Ser^518^ residues in intact cells. Consequently, the phosphorylation of JAK1 prevented the STAT3-dependent gene expression, e.g., the IL-6-induced responses. The inhibitors of JAK kinases, i.e., jakinibs, are claimed to be promising drugs in inflammatory and autoimmune diseases [79]. Interestingly, IL-6 and many other cytokines are potent inducers of the development as well as the activation of MDSCs (see above). There are also other AMPK-dependent mechanisms which can suppress the JAK-STAT pathways, e.g., AMPK activation can attenuate the nuclear translocation of STAT1 and thus inhibit the INF-γ-induced signaling [80]. Nerstedt et al. [81] demonstrated that the activation of AMPK by AICAR and metformin inhibited the IL-6-induced phosphorylation of STAT3. It needs to be recognized that AMPK signaling can also inhibit STAT3 factor through indirect pathways, e.g., by activating SIRT1 [82] which is a well-known target of AMPK signaling. Moreover, He et al. [83] reported that AMPK activation suppressed STAT1 signaling by increasing the expression of mitogen-activated protein kinase phosphatase-1 (MKP-1). Currently, it is known that AMPK activators can alleviate many chronic diseases, e.g., rheumatoid arthritis and chronic infections, which are associated with an overstimulation of the JAK-STAT signaling [84, 85]. Considering that MDSCs induce immunosuppression in chronic inflammatory diseases, it seems likely that the activation of AMPK signaling could alleviate the MDSC-induced immunosuppression by inhibiting the JAK-STAT signaling.

### NF-κB system

The NF-κB signaling pathway does not only regulate the immune responses but it also controls the development of myeloid cells, e.g., MDSCs and Tregs [86, 87]. There is convincing evidence that many inflammatory mediators, e.g., IL-1β, IL-6, and TNF-α, can augment the differentiation of MDSCs and stimulate their immunosuppressive properties (see above). Moreover, some alarmins, e.g., HMGB1 and S100A8 and A9, have been reported to enhance the immune suppressive properties of MDSCs in inflamed tissues [88, 89]. All these inflammatory factors are potent activators of the NF-κB pathway, which indicates that NF-κB signaling has an important role in the expansion of MDSCs in inflamed tissues. NF-κB signaling also has a crucial role in tumor initiation and progression [90]. Bunt et al. [91] demonstrated that the pro-inflammatory factors released by tumors induced the accumulation and activation of MDSCs, thus enhancing immune suppression and the immune escape of tumors. Recently, Flores et al. [63] revealed that the numbers of MDSCs significantly increased with aging in mouse bone marrow and spleen through the NF-κB-dependent mechanism. Given that MDSCs display a robust level of NF-κB activity, it seems that NF-κB signaling also induces the expansion of MDSCs during the inflammaging process. Moreover, Zhang et al. [92] reported that NF-κB signaling stimulated the differentiation of MDSCs into osteoclasts which then evoked bone erosion in collagen-induced mouse arthritis. Currently, the signaling pathways of NF-κB involved in the activation of MDSCs need to be clarified. It seems that the Toll-like receptor 4 (TLR4)/myeloid differentiation factor 88 (Myd88) signaling pathway is the major enhancer of the immunosuppressive activities of MDSCs and consequently induces immunosuppression, e.g., in conditions such as infections and cancers [93, 94]. Wang et al. [95] demonstrated that different TLR agonists specifically altered the differentiation and functions of human monocytic MDSCs. However, TLRs are linked not only to NF-kB signaling but some other pathways are also activated [96]. There is also cooperation with the NF-κB and STAT3 pathways linking inflammation to the development of several cancers [97]. For instance, the noncanonical NF-κB signaling pathway stimulated the STAT3-mediated expression of IDO in the MDSCs isolated from human breast cancer [74].

There is abundant literature indicating that the activation of AMPK inhibits NF-κB signaling via different mechanisms, e.g., by activating SIRT1, FoxO, p53, and PGC-1α signaling pathways [6]. AMPK can also prevent the activation of NF-κB signaling by inhibiting oxidative and endoplasmic reticulum (ER) stresses, well-known activators of NF-κB system. On the other hand, the activation of AMPK inhibited the TLR4/Myd88 pathway and attenuated immune responses [98, 99]. For instance, it was observed that AMPK activation reduced the expression of Myd88. Hong et al. [93] reported that the Myd88^−/−^ mice displayed a clear downregulation of the immunosuppressive properties in MDSCs. Recently, it was reported that AMPK activation regulates the functions of the NLRP3 subset of inflammasomes in both the aging process and tumorigenesis [100]. Inflammasomes are large multiprotein complexes which induce the maturation of the proforms of IL-1β (pro-IL-1β) and IL-18 (pro-IL-18) and subsequently activate distinct inflammatory cascades. The NF-κB system and NLRP3 inflammasomes undergo crucial cooperation since the NF-κB-dependent signaling induces the expression of NLRP3 as well as those of pro-IL-1β and pro-IL-18 [101]. Interestingly, in their studies conducted in transgenic NLRP^−/−^ mice, van Deventer et al. [102] demonstrated that NLRP3 inflammasomes had a critical role in the functions of MDSC, e.g., they increased the accumulation of MDSCs into tumors and enhanced their immune suppressive properties. Recently, Chen et al. [103] reported that the pharmacological inhibition of NLRP3 inflammasomes suppressed the accumulation of MDSCs, Tregs, and TAMs into head and neck squamous cell carcinoma (HNSCC) in mice and consequently, the anti-cancer immunity of CD4^+^ and CD8^+^ T cells was improved. Many cellular stresses, e.g., ER and mitochondrial stresses, stimulate NF-κB signaling and activate NLRP3 inflammasomes, whereas the activation of AMPK enhances the maintenance of homeostasis, e.g., by activating autophagy and suppressing immune responses through the inhibition of NF-κB signaling [6, 100, 104]. This indicates that AMPK activators could be promising drugs for the future treatment of cancers and age-related degenerative diseases.

### C/EBPβ factor

The CCAAT/enhancer-binding protein β (C/EBPβ) is a versatile transcription factor which has important functions in cellular differentiation, e.g., monocytic lineage determination [105], as well as in the regulation of ER stress and metabolism [106]. Marigo et al. [107] demonstrated that the stimulation of the immunosuppressive properties of both mouse bone marrow–derived and the tumor-provoked MDSCs were dependent on the function of C/EBPβ factor. They also reported that the deletion of the *Cebpβ* gene from the cells of hematopoietic lineage decreased the numbers and tolerogenic activity of MDSCs in both the spleen and tumor sites of tumor-bearing mice. There was a significant decrease in the protein levels and activities of ARG1 and NOS2 in the MDSCs infiltrated into tumors of the *Cebpβ-*deficient mice. Sepsis is another disease model in which C/EBPβ has a crucial role in the generation of immunosuppressive MDSCs in mouse bone marrow [108]. Dai et al. [108] demonstrated that the late phase of sepsis increased the expression of C/EBPβ protein which consequently induced the expression of miR-21 and miR181b in myeloid progenitors. This process stimulated the NF-κB signaling which enhanced myelopoiesis and expanded the population of immunosuppressive MDSCs. The conditional deletion of *Cebpβ* in myeloid cells reversed the development of MDSCs during sepsis. McPeak et al. [109] reported that the myeloid-specific deficiency of C/EBPβ protein increased the maturation of mouse myeloid cells rather than promoting the accumulation of immature MDSCs during the late phase of sepsis, thus reducing sepsis mortality. Interestingly, the C/EBPβ factor stimulated the expression of immunosuppressive TGF-β1 cytokine, whereas the TGF-β1-activated Smad3 and Smad4 factors repressed the transcriptional activity of C/EBPβ [110, 111]. These studies indicate that the regulation of C/EBPβ expression has a crucial role in the development and activity of MDSCs and thus it seems to be a promising drug target in many pathological conditions.

Given that adipogenesis is an anabolic process, it is known that AMPK signaling inhibits the differentiation of pre-adipocytes [112]. There is extensive evidence that the activators of AMPK can inhibit the expression and activity of C/EBPβ, a major inducer of adipogenesis, in pre-adipocytes and thus suppress adipocyte differentiation [113, 114]. It seems that AMPK signaling inhibits the C/EBPβ factor through indirect mechanisms. AMPK signaling represses the activity of C/EBPβ not only in adipocytes but it can also suppress diverse functions driven by C/EBPβ, e.g., AMPK inhibits the C/EBPβ-driven ER stress in hepatoma cells [115] and cardiomyocytes [116]. Matsuda et al. [117] demonstrated that ER stress in the mouse pancreatic islets induced the expression of C/EBPβ factor which consequently reduced the activity of AMPK, whereas the activation of AMPK suppressed the expression of C/EBPβ and alleviated ER stress. Lee et al. [118] reported that the ER stress, induced by thapsigargin, enhanced the immunosuppressive activity of MDSCs in tumor microenvironment. The expression of ARG1, iNOS, and NOX2 was significantly increased in tumor-infiltrated MDSCs. The administration of 4-phenylbutyric acid (4-PBA), an ER stress-reducing chaperone, substantially decreased the immunosuppressive capacity of MDSCs and restored antitumor immunity.

### CHOP factor

The C/EBP homologous protein (CHOP) is a transcription factor which is the main inducer of apoptosis in the conditions of ER stress [119]. Apoptosis is one of the mechanisms which regulate the number of MDSCs in inflamed tissues and thus control the level of immunosuppression in tissues. Chornoguz et al. [120] revealed that inflammatory conditions increased the resistance of MDSCs to apoptosis and thus enhanced their expansion in inflamed tissues. Thevenot et al. [121] demonstrated that MDSCs displayed the highest expression of CHOP in the sites of different mouse tumors as compared to other cell populations or even those of MDSCs in the spleen. The increased CHOP level in MDSCs correlated with their ability to inhibit T cell proliferation. The CHOP-deficient MDSCs displayed decreased immuno-regulatory functions, e.g., allowed the priming of T cell responses, enabled T cell proliferation, and delayed tumor growth. The exposure of ROS and peroxynitrite induced the expression of CHOP in mouse MDSCs, whereas antioxidants reduced its expression. Thevenot et al. [121] also reported that the expression of activating transcription factor 4 (ATF4), a key factor of integrated stress response (ISR), stimulated the expression of CHOP in MDSCs. Their results also indicated that the increased expression of CHOP activated the function of C/EBPβ/IL-6 axis which enhanced the immunosuppressive activity of MDSCs in inflamed tumor milieu. Recently, Shang et al. [122] revealed that mouse MDSCs expressed retinal non-coding RNA3 (RNCR3) which interacted with miR-185-5p, preventing its function. They demonstrated that miR-185-5p directly targeted the CHOP mRNA and thus blocked the translation of CHOP protein. The expression of RNCR3 in MDSCs was clearly increased in tumor microenvironment, probably stimulated by inflammatory cytokines. Shang et al. [122] also reported that the knockdown of RNCR3 in mice suppressed the differentiation and immunosuppressive properties of MDSCs which indicates that CHOP has an important role in the regulation of MDSC functions.

There is substantial evidence that the activation of AMPK can attenuate ER stress and protect tissues in diverse stress conditions [123, 124]. There probably exist different mechanisms through which AMPK can alleviate the detrimental effects of ER stress. Dai et al. [125] isolated mouse macrophages and demonstrated that AMPKα1 phosphorylated CHOP protein at Ser30 inducing its ubiquitination and subsequently its degradation by proteasomes. The deletion of *Ampkα1* gene significantly promoted the apoptosis of macrophages. These studies indicate that AMPK can prevent the overexpression of CHOP and thus counteract apoptotic cell death (Fig. 1). However, as revealed by Thevenot et al. [121] (above), the expression of CHOP was associated with an increased immunosuppressive capacity of MDSCs and thus it seems that MDSCs can evade apoptosis through the expression of survival factors induced by the ER stress-related activation of ATF4 [126].

### HIF-1α signaling

Tumors established a hypoxic microenvironment which stimulates the expression of hypoxia-inducible factors (HIF) in both tumor cells and immune cells [127]. Hypoxia promotes the accumulation of immunosuppressive cells, e.g., MDSCs and Tregs, into tumors, thus allowing tumor cells an opportunity for immune escape. HIF-1α signaling has crucial effects on both innate and adaptive immunity, e.g., HIF-1α promotes the recruitment of Tregs into tumors, increases the differentiation of Th17 cells, and activates the functions of dendritic cells [127, 128]. Corzo et al. [129] observed that hypoxia in the tumor milieu increased the expression of ARG1 and iNOS in mouse MDSCs and interestingly, hypoxia induced the conversion of tumor-infiltrated MDSCs into TAMs. TAMs are immunosuppressive macrophages since they express a high level of ARG1, iNOS, IL-6, and IL-10 and inhibited the proliferation of T cells. Noman et al. [130] reported that HIF-1α, but not HIF-2α, increased the expression of membrane-bound programmed death receptor ligand 1 (PD-L1), an immune checkpoint receptor, which mediates the inhibition of T cells by binding to the PD-1 receptor of T cells. Recently, Li et al. [131] demonstrated that the TGF-β-mTOR-HIF-1α signaling pathway induced the expression of CD39 and CD73 in human MDSCs. CD39 and CD73 are surface ectonucleotidases which hydrolyze extracellular ADP/ATP yielding adenosine. Ryzhov et al. [132] demonstrated that the adenosinergic regulation through the A2B receptors stimulated the proliferation and immunosuppressive activity of mouse granulocytic MDSCs. These studies indicate that hypoxia/HIF-1α signaling is a potent inducer of immunosuppression by enhancing the expression of immune suppressive factors in MDSCs.

Although AMPK and HIF signaling pathways are evolutionarily conserved host defense mechanisms, it does seem that they have many mutually antagonistic activities in the regulation of energy metabolism and immune responses [133]. However, AMPK has a close connection with HIF-1α signaling through several cooperative factors, e.g., sirtuin 1 (SIRT1), mammalian target of rapamycin (mTOR), and NF-κB signaling pathways which either activate or inhibit the function of HIF-1α. It has been recognized that the activation of AMPK stimulates the signaling of SIRT1, whereas it inhibits the functions of mTOR and NF-κB [6, 14, 16]. It is known that all these factors are able to enhance the function of HIF-1α via several mechanisms [134–136]. In addition, Chen et al. [137] demonstrated that AMPK regulated the nuclear localization of HIF-1α via the control of cytosolic shuttling of histone deacetylase 5 (HDAC5). It seems that the activation of AMPK can also inhibit the expression of immunosuppressive proteins induced by HIF-1α, e.g., CD39/CD73 and PD-L1. Li et al. [138] demonstrated that the metformin-induced AMPK activation reduced the expression of CD39 and CD73 in the MDSCs of ovarian cancer patients and consequently attenuated the immunosuppressive activity of MDSCs. Recently, Cha et al. [139] revealed that the metformin-activated AMPK directly phosphorylated PD-L1 protein at S195 which induced the abnormal glycosylation of PD-L1 and its subsequent degradation through the ER-associated protein proteasomal degradation (ERAD). These observations indicate that AMPK signaling can either enhance or suppress the HIF-1α-induced functions of MDSCs in a context-dependent manner (Fig. 1).

### NFE2L2/NRF2 signaling

Nuclear factor (erythroid derived 2) like (NFE2L2/NRF2) has not only a crucial role in the protection against oxidative stress but it also regulates innate immunity, e.g., through the inhibition of NF-κB signaling [140]. Since it is known that NFE2L2 is a powerful survival factor, it is not surprising that the activation of AMPK signaling stimulates the function of NFE2L2 pathway [141, 142] (Fig. 1). Joo et al. [142] observed that AMPK activation increased the accumulation of NFE2L2 transcription factors into the nuclei of HepG2 cells. They demonstrated that AMPK phosphorylated the Ser558 residue (Ser550 in mouse) in the human NFE2L2 protein. The mutation of these serine residues inhibited the accumulation of NFE2L2 into nuclei after AMPK activation. Because the Ser550/Ser558 residues are located in the nuclear export signal of NFE2L2 protein, it was proposed that the AMPK-induced phosphorylation prevented the export of NFE2L2 from the nuclei, thus enhancing the NFE2L2-mediated transcription. Beury et al. [143] compared the properties of MDSCs isolated from the NFE2L2^+/+^ mice and the transgenic NFE2L2^−/−^ mice. They reported that the MDSCs isolated from the NFE2L2^+/+^ mice suppressed more efficiently the function of CD4^+^ and CD8^+^ T cells than the MDSCs from the NFE2L2^−/−^ mice. In addition, to increased immune suppressive activity, the numbers of MDSCs in tumors were significantly higher in mice with NFE2L2^+/+^ in comparison with their counterparts deficient of NFE2L2. They also observed that the level of ROS compounds was significantly lower in the MDSCs with NFE2L2^+/+^ than in those lacking NFE2L2. This difference prevented the apoptosis of MDSCs since the tumor microenvironment is characterized by oxidative stress. Recently, Ohl et al. [144] revealed that the constitutive activation of NFE2L2 in mice induced the accumulation of MDSCs into the spleen. Moreover, the permanent increase in NFE2L2 activity enhanced the immune suppressive properties of MDSCs, e.g., mediated protection against LPS-induced sepsis. It seems that AMPK signaling activates the NFE2L2 pathway to facilitate the survival of MDSCs in diverse stresses, e.g., oxidative stress in inflammatory conditions (Fig. 1).

### AMPK controls MDSC activity via metabolic regulation

Currently, it has been recognized that energy metabolism is an important regulator of immune responses; the term immunometabolism has been coined to describe this process [145]. For instance, metabolic regulation has a crucial role in the functions of myeloid-derived immune cells in cancer and chronic inflammatory diseases [146, 147]. The Warburg effect, i.e., the aerobic glycolytic process producing lactate from glucose, is a well-characterized hallmark of cancer cells [148]. However, the Warburg effect does not only occur in tumors but recent studies have also revealed that immune cells in inflammatory diseases undertake energy production through aerobic glycolysis [149–151]. The activation of the myeloid cells, e.g., neutrophils, dendritic cells, and macrophages, stimulates aerobic glycolysis and thus triggers lactate production in inflamed tissues. Moreover, Menk et al. [152] demonstrated that the activation of the T cell receptor (TCR) induced the activation of pyruvate dehydrogenase kinase 1 (PDHK1) which inhibited pyruvate import into the mitochondria, thus facilitating its processing into lactate in the cytoplasm. Aerobic glycolysis has an important role in the shaping of T cell differentiation [153]. Lactate is not only a metabolite but it also possesses many signaling properties [154] and furthermore, it is a potent inducer of immunosuppression in inflammatory conditions, e.g., in sepsis [155]. For instance, in murine tumor models, the myeloid-specific knockout of lactate dehydrogenase-A (LDH-A) [156] or the reduction of lactate production by diclofenac exposure [157] reduced the T cell immunosuppression and increased antitumor immunity. Interestingly, Husain et al. [158] demonstrated that the addition of lactate to MDSC cultures significantly increased the expansion of immunosuppressive MDSCs which reduced the proliferation of CD4^+^ T cells and inhibited the cytotoxicity of CD8^+^ T cells and natural killer (NK) cells. Recently, Cai et al. [159] observed that the Epstein–Barr virus (EBV) promoted the proliferation of MDSCs in human nasopharyngeal carcinoma by increasing aerobic glycolysis and lactate production in cancer cells. The lactate shuttling between cells increases in the microenvironments of tumors and inflamed tissues [160] and it is possible that lactate acts as the inducer of expansion and activation of MDSCs in tissues and thus can augment tissue immunosuppression (Fig. 2).

Given that AMPK is a crucial metabolic regulator, it seems likely that AMPK can also control the functions of MDSCs through metabolic regulation. Interestingly, some transcription factors inhibited by AMPK signaling are potent enhancers of glycolysis, especially of the Warburg effect in immune cells (Fig. 2). It is known that STAT3 and NF-κB signaling pathways, suppressed by AMPK activation (Fig. 1), can stimulate HIF-1α signaling which is a major inducer of aerobic glycolysis and lactate production [149, 161]. However, AMPK signaling is also able to enhance the activity of HIF-1α, e.g., NFE2L2/NRF2 induces the metabolic shift to aerobic glycolysis via HIF-1α signaling [162]. Mammalian target of rapamycin (mTOR), inhibited by AMPK activation, is an effective activator of HIF-1α signaling and thus can trigger lactate production [134] (Fig. 2).

There is abundant evidence that mTOR is an important regulator of the differentiation and immunosuppressive functions of MDSCs [163–165]. Deng et al. [165] demonstrated that the mTOR-induced glycolysis enhanced the immune suppressive properties of tumor-infiltrated monocytic MDSCs in mice. In contrast, rapamycin treatment reduced the level of glycolysis and decreased the suppressive activities of MDSCs. Chen et al. [164] revealed that the isolated hepatic MDSCs from the mice with the Con A-induced immune-mediated hepatic injury (CIH) displayed an increased glycolytic activity along with increased expression of glycolytic enzymes, e.g., LDH-A. It was observed that rapamycin decreased the glycolytic activity in the hepatic MDSCs of CIH mice. Similar results were observed after the exposure of 2-deoxy-D-glucose (2-DG), a competitive inhibitor of glucose-6-phosphatase. The mTOR signaling in hepatic MDSCs controlled the HIF-1α-dependent glycolytic activity and suppressed T cell activation. Furthermore, Makki et al. [163] reported that the exposure of obese mice to rapamycin increased the total number of granulocytic MDSCs whereas that of monocytic MDSCs was decreased in both the blood and adipose tissue. It seems that metabolic regulation can differently affect the two subtypes of MDSCs as well as controlling the polarization of MDSCs between the pro-inflammatory M1 and immunosuppressive M2 phenotypes. For instance, it is known that sirtuin 1 (SIRT1), the cooperating partner of AMPK, regulates the M1/M2 switch of MDSCs through the mTOR-HIF-1α pathway [166]. These studies indicate that there is a complex regulatory network behind the glycolytic reprogramming of the suppressive properties of MDSCs.

Aerobic glycolysis is not the only source of lactate since glutaminolysis, i.e., the catabolism of L-glutamine can generate lactate in mouse MDSCs [167] (Fig. 2). In particular, the use of L-glutamine is an important carbon source in the maturation of MDSCs. It is known that glutaminolysis is a crucial compensatory energy pathway in conditions where the supply of pyruvate into mitochondria is compromised [168]. Recently, Morikawa et al. [169] demonstrated that the metabotropic glutamate receptor 2/3 (mGluR2/3) was expressed in mouse MDSCs and it played an important role in the maintenance of immunosuppressive properties of MDSCs. For instance, cancer cells secrete glutamate which can be processed in cells through glutaminolysis. Overall, it seems that the metabolic regulation of MDSC functions is controlled by the energy metabolic processes in both MDSCs and their neighboring cells.

Fatty acids are not only the source of energy production through β-oxidation but they have diverse functions in the regulation of immune system [170, 171]. For instance, there is substantial evidence that the dietary supplementation of polyunsaturated fatty acids (PUFA), i.e., omega-3 (ω-3) and omega-6 (ω-6) fatty acids, have immunosuppressive responses, e.g., to the signal transduction and antigen presentation of human T cells [172, 173]. Moreover, ω-3 and ω-6 fatty acids are the precursors of prostaglandin synthesis via cyclooxygenases (COX) and it is known that prostaglandin E2 (PGE2) exerted profound effects on immune cells [174]. Interestingly, Yan et al. [72] demonstrated that both the ω-3 and ω-6 PUFA treatments significantly enhanced the expansion of cultured bone marrow MDSCs, especially that of granulocytic MDSCs. The proliferation of T cells decreased in a dose-dependent manner of PUFAs. The exposure of mice with PUFAs increased the percentage of granulocytic MDSCs in both the bone marrow and the spleen. The administration of PUFA also stimulated the immunosuppressive properties of MDSCs isolated from mouse spleen. The PUFA treatment induced the activation of JAK-STAT3 signaling (Fig. 2) and the immunosuppression of T cells was mediated by ROS. Finally, Yan et al. [72] revealed that a PUFA-enriched diet augmented the growth of CT26 and Lewis lung carcinoma in mice. In agreement with these results, Xia et al. [175] reported that provision of a ω-3 enriched fish oil diet promoted the expansion of MDSCs in the mouse spleen. Correspondingly, this diet suppressed the cytotoxicity of CD8^+^ T cells and increased the growth of melanoma. Given that PUFAs activate PGE2 production through COX-2 (above), it is interesting that PGE2 can stimulate STAT3 signaling [176]. It is known that PGE2 is a potent inducer of the functions of MDSCs [177] and thus might stimulate the expansion of MDSCs. Remarkably, it seems that AMPK signaling can suppress the PUFA-induced expansion of MDSCs since it is known that in addition to the JAK-STAT3 signaling, AMPK activation can also inhibit the COX-2 signaling [178]. Moreover, the inhibition of COX-2 enzyme by celecoxib administration reduced the level of PGE2 and prevented the expansion of both MDSC subtypes in tumor-bearing mice [179]. These studies indicate that AMPK signaling is a potent inhibitor of the PUFA-induced activation of MDSCs (Fig. 2).

Obesity is associated with chronic inflammation in adipose tissues and thus it is not surprising that MDSCs accumulate into adipose tissues during obesity [180]. It is known that the fatty acid oxidation (FAO) of MDSCs increases in the tumor microenvironment [181, 182]. Hossain et al. [181] demonstrated that the inhibition of FAO after exposure to etomoxir or ranolazine reduced the immunosuppressive properties of MDSCs, e.g., the expression of ARG1 and several cytokines as well as their ability to inhibit T cell proliferation. The inhibition of FAO also suppressed the growth of carcinomas in mice. In addition, they reported that the capacity of fatty acid uptake and oxidation was significantly increased in the circulating and tumor-infiltrated MDSCs in cancer patients. The mechanisms underpinning the FAO-induced activation of MDSCs still need to be clarified (Fig. 2). However, Al-Khami et al. [182] demonstrated that the tumor-derived factors, e.g., GM-CSF and IL-6, stimulated lipid uptake and metabolism in tumor-infiltrated MDSCs. An increase in lipid oxidation enhanced the immunosuppressive properties of MDSCs through the activation of STAT3 signaling in the tumor microenvironment. They also observed that the deletion of CD36, a fatty acid translocase, decreased the immunosuppressive properties of MDSCs and inhibited tumor growth in CD36 knockout mice. Interestingly, it has been reported that CD36 protein inhibits the activation of AMPK but the binding of fatty acid to the CD36 receptor activates AMPK signaling which consequently stimulates the β-oxidation of fatty acids [183] (Fig. 2). One might speculate that the activation of CD36 by fatty acids stimulates AMPK signaling which enhances energy production through FAO while at the same time, it might control the immunosuppressive functions of MDSCs via an inhibition of major inducers of MDSCs (Fig. 1).

### AMPK-mediated control of MDSCs in cancer therapy and longevity regulation

There is substantial evidence that AMPK signaling regulates cancer growth although many studies have revealed that AMPK activation can induce either an oncogenic or a tumor suppressive process in a context-dependent manner [5]. It seems that AMPK signaling can act as a tumor suppressor by inhibiting the function of major transcription factors associated with tumorigenesis in several ways, e.g., (i) AMPK inhibits the AKT/mTOR-dependent cancer growth [184], (ii) AMPK inhibits the JAK-STAT signaling which enhances tumorigenesis [185], (iii) AMPK inhibits the NF-κB system linked to the inflammation-mediated tumorigenesis [90], and (iv) AMPK inhibits C/EBPβ signaling which affects metastasis [186]. AMPK activation can also context-dependently inhibit HIF-1α signaling which is a crucial inducer of carcinogenesis [187]. Interestingly, all of these pathways are important enhancers of MDSC expansion and activation in inflamed microenvironment suggesting that the activators of AMPK might be important drugs in cancer therapy. Currently, there is clear evidence that metformin, an AMPK activator, is a promising drug candidate for cancer therapy although metformin also has AMPK-independent targets [188]. Uehara et al. [69] demonstrated that metformin administration inhibited the growth of osteosarcoma in mice. They observed that metformin treatment reduced the numbers of granulocytic MDSCs in both the spleen and tumors. Metformin treatment increased the ROS production of isolated MDSCs and redirected their energy metabolism from oxidative phosphorylation to glycolysis. In addition, many flavonoids and terpenoids might mediate the inhibition of MDSCs through the activation of AMPK signaling [53]. There are several possible mechanisms to explain how the activators of AMPK could suppress the functions of MDSCs in cancer therapy. For instance, it is known that AMPK activators downregulate the chemokine signaling in macrophages [189, 190] which might impair the chemokine-driven accumulation of MDSCs into tumors. Given that AMPK activators have displayed beneficial effects in cancer combination therapies [191], it would be important to clarify whether they could improve cancer immunotherapies by inhibiting the MDSC-induced immunosuppression.

AMPK signaling has a crucial role in the regulation of longevity since it controls several signaling pathways associated with the aging process [16, 17]. For instance, AMPK activation inhibits the NF-κB and mTOR-mediated signaling, two pathways known to accelerate the aging process. In contrast, AMPK signaling stimulates SIRT1, FoxO/DAF-16, and NFE2L2/NRF2 pathways which enhance stress resistance and can increase longevity. In addition, the signaling of two pro-longevity factors, i.e., caloric restriction [192] and fibroblast growth factor 21 (FGF21) [193], stimulate AMPK signaling and thus could affect the regulation of MDSCs. Currently, there is no direct evidence on the role of caloric restriction and FGF21 in the activation or expansion of MDSCs. However, metformin is a promising tool to delay the aging process [194] and it inhibits the expansion of MDSCs [69]. As described above, the presence of MDSCs significantly increases with aging, probably induced by the chronic inflammaging condition in tissues. It seems that AMPK signaling can influence the appearance and activity of MDSCs in a multifaceted manner. For instance, AMPK activation prevents inflammatory responses by inhibiting the NF-κB signaling system and thus it downregulates the generation of chemotactic factors in inflamed tissues which consequently eliminates the generation of MDSCs in the bone marrow. In addition, it is known that the activation of mTOR, a well-known longevity suppressor, stimulates the expression of G-CSF which increases the expansion and infiltration of MDSCs into tumors [195]. AMPK signaling can also directly control the differentiation of myeloid cells [196] which might affect the generation of MDSCs with aging. On the other hand, AMPK signaling can suppress the major signaling pathways which stimulate the immunosuppressive properties of MDSCs (Fig. 1) and thus it could exert a local control of the functions of MDSCs. Given that AMPK signaling promotes catabolic activities rather than anabolic processes, it is likely that AMPK activation suppresses energy-consuming translational processes and thus inhibits the proliferation of MDSCs. Currently, the role of MDSC expansion associated with inflammaging needs to be clarified although it seems that the age-related immunosenescence could be induced by the activation of immunosuppressive network involving MDSCs, Tregs, and regulatory M2 macrophages [11]. Immunosuppressive cells secrete anti-inflammatory cytokines, e.g., TGF-β and IL-10, which have degenerative bystander effects in inflamed tissues [10]. Moreover, the increased expression of ARG1 and IDO induces a shortage of amino acids disturbing the maintenance of proteostasis in aged tissues. The age-related expansion of MDSCs could also increase the prevalence of tumors and chronic infections and reduce the efficacy of cancer immunotherapies. It seems that the activators of AMPK signaling are potent inhibitors of MDSC functions and subsequently, they might mitigate the effects of inflammaging and thus extend healthspan and lifespan.

## Conclusions

AMPK signaling has several functions beyond the regulation of energy metabolism. For instance, AMPK signaling regulates many activities of innate and adaptive immunity by targeting the major immune signaling pathways which control the differentiation and activity of immune cells. Moreover, AMPK signaling regulates the energy metabolism of immune cells and these immunometabolic processes have a crucial role in the control of activity of immune cells. It seems that AMPK activation regulates the functions of MDSCs in a context-dependent manner. Given that MDSCs have a key role in the maintenance of immunosuppression in conditions of chronic inflammation, the AMPK-induced suppression of MDSC expansion and activation might increase the efficiency of the immune system, e.g., in tumors and chronic infections, and thus alleviate chronic inflammation. In addition, AMPK activation might also mitigate age-related immune deficiency and thus improve healthspan. AMPK signaling also controls the energy metabolism of MDSCs by enhancing the fatty acid oxidation which increases the immunosuppressive potentials of MDSCs. Metabolites can also control the activity of MDSCs since lactate stimulates the immunosuppressive properties of MDSCs. Lactate could be an important inducer of the functions of MDSCs during acute inflammation and thus enhance the resolution of inflammation. It is known that immune cells switch on aerobic glycolysis during acute inflammation. Moreover, tumor cells display increased aerobic glycolysis associated with the production of lactate which might be an important enhancer of the expansion and activation of MDSCs in tumor sites.
